# Beyond the Beam: Multimodal Imaging and Surveillance of Post-Radiotherapy Changes in the Breast

**DOI:** 10.3390/life16040701

**Published:** 2026-04-21

**Authors:** Silvia Gigli, Giacomo Bonito, Emanuele David, Corrado Spatola, Brandon M. Ascenzi, Roberta Valerieva Ninkova, Sandrine Riccardi, Lucia Malzone, Paolo Ricci, Lucia Manganaro

**Affiliations:** 1 Department of Diagnostic Imaging, Sandro Pertini Hospital, Via dei Monti Tiburtini 385, 00157 Rome, Italy; 2 Department of Emergency Radiology, Policlinico Umberto I Hospital, Sapienza University of Rome, Viale del Policlinico 155, 00161 Rome, Italy; 3 Radiology Unit 1, Department of Medical Surgical Sciences and Advanced Technologies “GF Ingrassia”, University Hospital “Policlinico G. Rodolico”, University of Catania, 95123 Catania, Italy; 4 Radiotherapy Unit, Department of Medical Surgical Sciences and Advanced Technologies “GF Ingrassia”, University Hospital Policlinic “G. Rodolico-San Marco”, 95123 Catania, Italy; 5 Independent Neuroresearcher, Member of Marie Curie Alumni Association (MCAA), 03012 Anagni, Italy; 6 Department of Radiological, Oncological and Pathological Sciences, Policlinico Umberto I Hospital, Sapienza University of Rome, Viale Regina Elena 324, 00161 Rome, Italy

**Keywords:** breast cancer, radiotherapy, post-radiotherapy changes, imaging surveillance, mammography, ultrasound, MRI, nuclear medicine, fibrosis, fat necrosis, local recurrence

## Abstract

Breast-conserving therapy, consisting of lumpectomy followed by adjuvant radiotherapy, is the standard of care for early-stage breast cancer, providing oncologic outcomes equivalent to mastectomy while preserving breast anatomy and quality of life. Radiotherapy remains a cornerstone of treatment across disease stages, significantly reducing local recurrence rates and improving long-term survival. Advances in radiotherapy techniques—including conventional fractionation, hypofractionation, tumor-bed boost delivery, and regional nodal irradiation—have optimized oncologic efficacy while inducing a broad spectrum of time-dependent morphological changes in breast tissue. Accurate imaging surveillance is therefore essential to distinguish expected post-radiotherapy changes from tumor recurrence and to avoid unnecessary diagnostic or therapeutic interventions. This review provides a comprehensive overview of contemporary breast radiotherapy protocols, their impact on post-treatment imaging appearances, and current recommendations for imaging surveillance. Characteristic findings across mammography, ultrasound, magnetic resonance imaging, and nuclear medicine modalities are discussed, with emphasis on their temporal evolution from acute inflammatory changes to chronic fibrosis, fat necrosis, and architectural distortion. Recognition of these imaging patterns, together with integration of radiotherapy-related parameters into image interpretation, is crucial for accurate diagnosis, early detection of recurrence, and informed clinical management of breast cancer survivors.

## 1. Introduction

Breast cancer remains one of the leading causes of cancer-related morbidity and mortality among women worldwide, despite substantial advances in early detection and multimodal treatment strategies [1]. Global epidemiological data confirms a steadily increasing incidence, accompanied by significant improvements in survival due to optimized screening programs and advances in systemic and locoregional therapies [1,2].

Breast-conserving therapy (BCT), consisting of lumpectomy followed by adjuvant radiotherapy, has become the standard treatment for a substantial proportion of patients with early-stage breast cancer. Multiple randomized trials and long-term follow-up studies have demonstrated that BCT provides overall survival rates equivalent to mastectomy, while offering superior cosmetic outcomes and improved quality of life [3,4].

Radiotherapy (RT) plays a pivotal role in breast cancer management across different disease stages, including early, locally advanced, and selected metastatic settings. In the adjuvant setting, RT significantly reduces the risk of ipsilateral breast tumor recurrence and contributes to long-term reductions in breast cancer-specific mortality, as demonstrated by large individual patient–data meta-analyses [5].

Over the past two decades, breast radiotherapy has undergone major technical and conceptual evolution. Traditional fractionation methods have increasingly been supplemented—and sometimes substituted—by approaches like hypofractionated regimens, partial breast irradiation, tumor-bed boost strategies, and regional nodal irradiation protocols. These techniques let clinicians tailor treatment according to tumor biology, patient traits, and surgical choices [6,7,8].

While these advances have optimized oncologic outcomes and patient convenience, they have also introduced a broad spectrum of post-treatment imaging findings. The appearance, distribution, and temporal evolution of radiation-induced changes depend on multiple factors, including total dose, dose per fraction, irradiated volume, and time elapsed since treatment [9]. These changes may mimic or obscure tumor recurrence, representing a frequent diagnostic challenge during imaging surveillance [10].

Accurate interpretation of post-radiotherapy breast imaging therefore requires familiarity with expected treatment-related changes and their chronological progression. International guidelines emphasize structured imaging follow-up after radiotherapy, with modality selection and timing adapted to the type of surgery and radiotherapy received [11,12]. Integrating clinical information and radiotherapy parameters into image interpretation is essential to minimize false-positive findings and avoid unnecessary invasive procedures.

The purpose of this narrative review is to: (i) summarize contemporary breast radiotherapy regimens and their clinical indications; (ii) review current recommendations for imaging surveillance after radiotherapy; and (iii) describe typical post-radiotherapy imaging findings across mammography, ultrasound, magnetic resonance imaging, and nuclear medicine modalities, highlighting features that aid in differentiating benign treatment-related changes from disease recurrence.

## 2. Methods

This is a narrative review focused on imaging appearances after breast radiotherapy and practical surveillance strategies. A targeted literature search was performed in PubMed and major society guidance documents (ASTRO, ESMO/ESTRO, AIRO, ACR, NCCN) for the period 2000–2025, prioritizing randomized trials of fractionation, guideline statements, and imaging reviews describing post-treatment findings across modalities. The literature search was performed using combinations of keywords including “breast cancer,” “radiotherapy,” “post-radiotherapy changes,” “breast imaging,” “mammography,” “ultrasound,” “MRI,” “magnetic resonance imaging,” “surveillance,” and “local recurrence.” Articles were selected based on their clinical relevance, methodological robustness, and contribution to the understanding of imaging features and temporal evolution of radiation-induced breast changes.

Additional classic pathology and imaging papers were included when repeatedly cited in contemporary reviews or considered important for contextual interpretation of post-treatment findings. Evidence was synthesized qualitatively, with emphasis on clinically actionable imaging patterns and time-dependent “expected vs. suspicious” changes that may assist radiologists in differentiating treatment-related alterations from tumor recurrence. This narrative review has inherent limitations, including the absence of a systematic search strategy and the potential for selection bias. However, its aim is to provide a practical, experience-based framework for image interpretation rather than an exhaustive quantitative synthesis.

## 3. Radiotherapy Regimens

Breast radiotherapy has evolved over recent decades, with modifications in dose, fractionation, target volumes, and treatment techniques aimed at improving oncologic outcomes, reducing toxicity, and enhancing patient convenience. A clear understanding of the main radiotherapy schedules is essential for imaging specialists, as treatment parameters directly influence the type, extent, and temporal evolution of post-radiotherapy imaging findings. Table 1 summarizes typical prescriptions, indications, practical advantages and limitations, and key imaging implications.

### 3.1. Whole-Breast Irradiation with Standard Fractionation

Conventional whole-breast irradiation (WBI) delivers a total dose of 45–50.4 Gy to the entire breast in 25–28 fractions of 1.8–2.0 Gy, typically administered over five to six weeks. This regimen has historically represented the standard of care following breast-conserving surgery and remains an option in selected clinical scenarios [13].

In many patients, a tumor-bed boost of 10–16 Gy is added, particularly in younger women or in those with high-risk pathological features, such as close or positive surgical margins. While standard fractionation provides excellent long-term local control, its prolonged treatment duration may negatively impact on quality of life and access to care, particularly for patients living far from radiotherapy centers [14].

### 3.2. Whole-Breast Irradiation with Hypofractionation

Hypofractionated WBI delivers a slightly lower total dose over fewer fractions, exploiting the low α/β ratio of breast cancer. Moderate hypofractionation typically consists of 40–42.5 Gy in 15–16 fractions (2.65–2.8 Gy per fraction) administered over approximately three weeks. Large, randomized trials, including the START A and B trials and the Canadian trial, have demonstrated non-inferiority of moderate hypofractionation compared with conventional fractionation in terms of local control, overall survival, and cosmetic outcomes [15,16].

Based on this robust evidence, moderate hypofractionation is now recommended as the preferred regimen for most patients undergoing whole-breast irradiation, regardless of age, breast size, or nodal status, according to international guidelines from ASTRO, ESMO, NCCN, and AIRO [7,8,11,13].

Ultra-hypofractionated regimens have further reduced treatment duration. The FAST-Forward trial demonstrated that a schedule of 26 Gy in five fractions over one week is non-inferior to 40 Gy in 15 fractions in terms of ipsilateral breast tumor recurrence at five years, with acceptable toxicity profiles [17]. This approach is increasingly adopted for both whole-breast and chest-wall irradiation in appropriately selected patients [18].

### 3.3. Partial Breast Irradiation

Partial breast irradiation (PBI) targets only the surgical cavity and a limited surrounding margin, based on evidence that most local recurrences occur near the original tumor bed. PBI can be administered through various methods, such as interstitial or intracavitary brachytherapy, external beam radiotherapy with smaller treatment areas, and intraoperative radiotherapy (IORT) [19].

Randomized trials such as ELIOT and IMPORT LOW have demonstrated that, in carefully selected low-risk patients, PBI provides local control rates comparable to WBI, with reduced treatment times and potentially improved cosmetic outcomes [20]. However, patient selection is critical, and PBI is reserved for older patients with small, low-grade, hormone receptor-positive tumors and negative lymph nodes.

### 3.4. Regional Nodal Irradiation and Post-Mastectomy Radiotherapy

Regional nodal irradiation (RNI) and post-mastectomy radiotherapy (PMRT) are indicated in patients with node-positive disease, high-risk pathological features, or locally advanced tumors. These approaches typically include irradiation of the chest wall and regional lymph node basins, such as the axillary, supraclavicular, and internal mammary nodes. Standard fractionation schedules of 45–50.4 Gy in 25–28 fractions have traditionally been used; however, moderate hypofractionation schedules have demonstrated comparable efficacy and safety and are increasingly considered standard of care. Modern radiotherapy techniques, including IMRT and VMAT, combined with breath-hold strategies, allow for improved target coverage while minimizing dose to critical organs such as the heart and lungs [21,22]. Recent evidence suggests that in selected patients who achieve a complete nodal response after neoadjuvant chemotherapy, omission of RNI may be safe, highlighting the evolving and individualized nature of contemporary radiotherapy decision making.

## 4. Surveillance Imaging After Radiotherapy: Practical Strengths and Limitations

Structured imaging surveillance is a fundamental component of breast cancer care after radiotherapy. The choice of imaging modality and the timing of follow-up examinations are influenced by the type of surgery, radiotherapy technique, and individual patient risk factors [9,10,11].

Surveillance imaging should be timed to balance early detection of recurrence with avoidance of false positives from acute inflammation. Most guidelines recommend the first post-treatment mammogram no earlier than 6 months after completion of RT (and often 6–12 months after surgery, depending on local pathways), followed by annual mammography [10,11]. Earlier targeted evaluation is appropriate for new symptoms (palpable mass, skin changes, persistent focal pain) or for imaging abnormalities that evolve rather than stabilize.

Mammography remains the reference standard after breast-conserving therapy, as it enables reproducible longitudinal assessment of architectural distortion and calcifications and provides a stable baseline for comparison; however, in the early post-radiotherapy phase its diagnostic performance is frequently limited by diffuse parenchymal edema, increased breast density, trabecular thickening, and skin thickening, which predominantly reflect inflammatory and vascular effects rather than residual disease [9,10].

In this setting, mammography primarily serves as a reference examination, while interval stability or regression on subsequent studies is a key indicator of benignity.

Ultrasound plays an important complementary role, particularly for targeted evaluation of palpable abnormalities or focal mammographic findings, offering real-time assessment of tissue echotexture and facilitating image-guided biopsy; nevertheless, considerable overlap persists between fibrotic change, fat necrosis, and recurrent tumor, underscoring the need for multimodality correlation [10].

Magnetic resonance imaging (MRI) provides high sensitivity in selected patients, especially those with dense breasts, extensive postsurgical distortion, or equivocal findings on conventional imaging, but its specificity is reduced within the first 6–12 months after radiotherapy due to treatment-related enhancement and edema, making careful temporal comparison essential for confident interpretation [23].

Positron emission tomography/computed tomography (PET/CT) has a limited role in routine local surveillance, as radiation-induced inflammatory FDG uptake frequently leads to false-positive findings early after treatment; however, it may contribute valuable information in the evaluation of suspected chest wall recurrence, nodal disease, or distant metastases in selected clinical scenarios [24]. Overall, effective post-radiotherapy surveillance relies on appropriate timing of imaging, integration of complementary modalities, and radiologist awareness of the temporal evolution of post-treatment changes to balance early detection of recurrence with avoidance of unnecessary interventions.

## 5. Imaging Appearance over Time: Expected Findings vs. Red Flags

A pragmatic approach is to interpret findings by time since RT, recognizing that the peak of edema/skin thickening commonly occurs within the first several months, while fat necrosis, dystrophic calcifications, and scar-related architectural distortion evolve over 6–24 months and then usually stabilize. The most important “red flag” is progression after stability: new or enlarging mass, increasing asymmetry/distortion, or developing suspicious calcification pattern, particularly beyond the first post-treatment year. A structured summary of expected findings and suspicious features according to time interval is provided in Table 2; the following sections focus on modality-specific interpretation and diagnostic nuances rather than reiterating tabulated features.

In the weeks following completion of radiotherapy, inflammatory and vascular changes within the treated breast commonly lead to diffuse edema, trabecular thickening, and skin thickening. These findings usually peak during the first months after treatment and gradually regress over time [25,26].

### 5.1. Breast Edema and Skin Thickening

Breast edema lacks a universally accepted definition and standardized assessment criteria. Reported clinical features include increased breast volume, peau d’orange appearance, heaviness, skin erythema, pain, and ptosis [27]. Unlike focal postoperative edema, radiation-induced edema typically involves the entire breast, with prominent periareolar involvement often related to surgical disruption of lymphatic drainage [28].

Skin thickening represents another consistent and well-recognized effect of radiotherapy, with reported incidence rates ranging from 74% to 100% among treated patients [26]. This reaction is primarily attributable to increased lymphatic flow and the resulting distension of lymphatic vessels and subcutaneous capillaries. Several factors have been associated with the development and severity of these cutaneous changes, including higher radiation doses, larger breast volumes, and the delivery of an additional boost to the tumor bed. In some cases, skin thickness may reach values of up to 1 cm [29].

**Figure 1:**
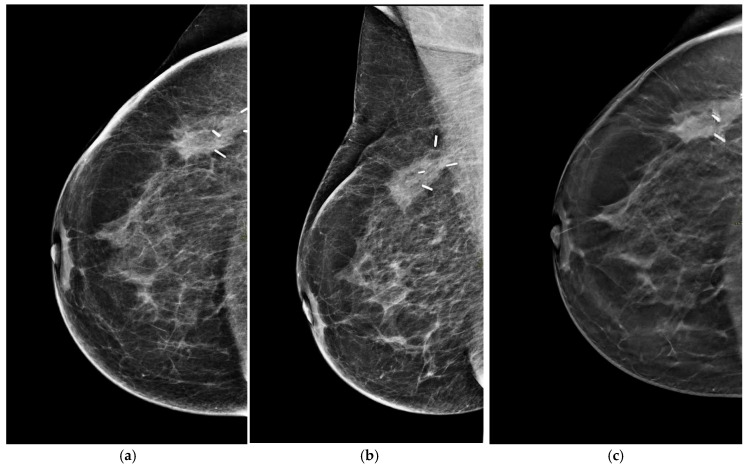
Six-month post-radiotherapy mammographic follow-up in a 56-year-old woman in axial (**a**) and oblique (**b**) projections, better visible in tomosynthesis. (**c**) shows skin thickening, breast edema, and increased density around the surgical clips, which corresponds on ultrasound to a fluid collection.

On mammography, breast edema typically manifests as increased breast density and accentuation of the trabecular pattern, often more evident near the surgical site. These findings should be interpreted in comparison with the contralateral breast and prior examinations, as their gradual regression over time strongly supports benign post-treatment change [30] (Figure 1a–c).

In a retrospective study involving 92 patients, an automated breast density segmentation tool was used to quantify changes in mammographic density before and after breast cancer treatment [31]. The results suggested that quantitative assessment of breast density may represent a useful tool for monitoring treatment-related changes over time.

As summarized in Table 2, these imaging findings generally decrease during follow-up. Conversely, an increase in breast edema or skin thickness after a documented period of stability should prompt careful reassessment and further diagnostic evaluation. The differential diagnosis of progressive breast edema includes lymphatic spread of breast cancer, impaired venous drainage, congestive heart failure, and infectious processes [31,32].

**Figure 2:**
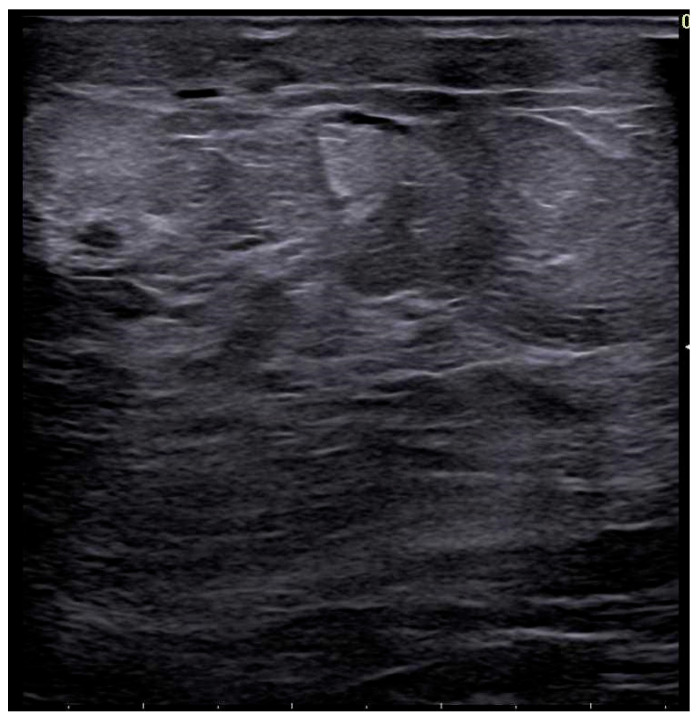
Corresponding ultrasound of post-radiotherapy skin thickening/edema.

Ultrasound (US) allows both objective assessment of skin thickness and qualitative evaluation of changes in tissue echogenicity, particularly when the treated breast is compared with the contralateral side (Figure 2). Breast edema on US appears as irregular, hypoechoic linear structures within the subcutaneous tissue, corresponding to dilated subdermal lymphatics or interstitial fluid accumulation. These findings must be carefully distinguished from ductal structures. Color Doppler imaging may demonstrate increased vascularity, reflecting the underlying inflammatory response [27].

A systematic review by Hussein et al. demonstrated that skin toxicity features in patients undergoing radiotherapy include skin thickening, reduced dermal echogenicity, and diminished contrast between the dermis and hypodermis when compared with non-irradiated skin [33]. In response to the need for more objective and reproducible assessment methods, Gao et al. developed an automated ultrasound-based skin segmentation technique capable of quantitatively evaluating radiation-induced changes in skin thickness. This approach has the potential to provide more consistent and clinically feasible measurements of tissue damage than the subjective assessments currently employed in routine practice [34].

Furthermore, a prospective longitudinal study involving 67 patients with breast cancer explored the role of ultrasound histogram analysis in objectively quantifying acute radiation-induced breast toxicity. Serial B-mode ultrasound examinations were performed at standardized anatomical sites on both the irradiated and contralateral breasts at four predefined time points: prior to radiotherapy, at completion of treatment, and at 3–4 and 9–12 weeks following therapy [35]. The authors demonstrated that quantitative ultrasound histogram analysis may serve as a reliable objective biomarker for monitoring acute radiation-induced breast toxicity, offering improved reproducibility and sensitivity compared with conventional clinical grading systems.

On MRI, radiation-induced breast changes on T2-weighted sequences, including edema, skin thickening, and seroma formation, closely resemble those observed on mammography (Figure 2). Jie Li et al. reported edema and skin thickening as the most common findings, detected in over 70% of patients at the first post-treatment MRI [36]. These alterations are typically most evident on the initial follow-up examination and gradually decrease over the subsequent three years after breast-conserving therapy, eventually stabilizing. However, T2-weighted edema may persist as a long-term finding in some patients beyond six years [36].

In nuclear imaging, FDG-PET/CT often shows increased uptake within irradiated tissues due to inflammatory changes, while Tc-99m sestamibi SPECT may demonstrate diffuse tracer accumulation in the acute phase [37]. Proper interpretation requires correlation with clinical history and treatment timing to prevent misdiagnosis and unnecessary biopsy.

### 5.2. Fluid Collections

**Figure 3:**
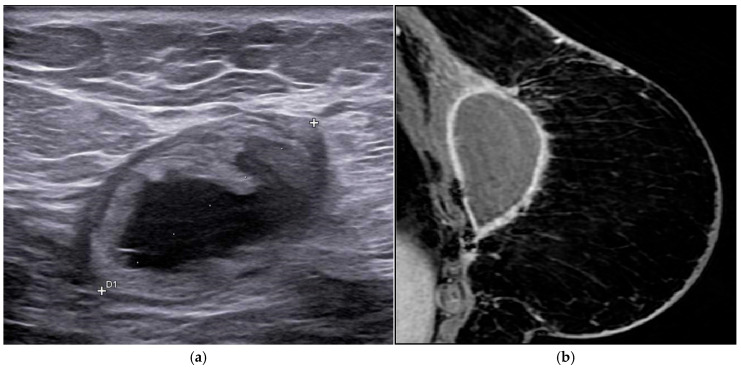
A 67-year-old woman, four months after fractionated radiotherapy, presents with a palpable sub-scar mass. Ultrasound evaluation shows a complex lesion with mixed solid–fluid components, without internal vascularity on color Doppler, consistent with an organizing fluid collection (**a**). MRI shows an extensive fluid collection with peripheral wall enhancement within the pectoralis muscle. The collection was partially drained and subsequently monitored with ultrasound follow-up, showing partial resolution over time (**b**).

Fluid collections, or seromas, are common early findings in patients who have undergone breast-conserving therapy. On mammography, these changes typically appear as oval or round masses and may mimic disease recurrence if interpreted in isolation. Ultrasound (US) plays a pivotal role in the differential diagnosis, demonstrating an anechoic or complex cystic mass with septations, loculations, thickened walls, or a combination of these features (Figure 3a) [38,39].

The internal composition and enhancement characteristics of seromas are variable. In most cases, the collection contains simple fluid; however, MRI may reveal the presence of blood products, fat, or a mixture of these components in some patients. On follow-up MRI examinations, seromas typically show a progressive reduction in size. Peripheral rim enhancement may be observed in the early post-treatment period and should not be misinterpreted as tumor recurrence at this stage (Figure 3b) [40]. In patients with fat-containing seromas, fat necrosis is usually present and is commonly associated with peripheral rim enhancement surrounding the collection.

Postoperative seromas and hematomas gradually decrease in size and are eventually replaced by scar tissue and fibrosis. Fluid collections regress over time and are completely resorbed within 12–18 months after surgery, although persistence may be observed in a minority of cases [39]. Any increase in the size of a fluid collection on serial imaging should prompt further evaluation, as it may indicate a superimposed complication or disease recurrence [39].

## 6. Long-Term Changes (Approximately Six Months to Two Years Post Radiotherapy)

The intermediate to chronic post-radiotherapy phase is marked by a gradual resolution of inflammation, accompanied by tissue remodeling and the development of fat necrosis and architectural distortion, which represent the imaging correlates of fibrosis. In this stage, adipocyte destruction leads to the release of lipases, with necrotic fat residues becoming encapsulated by fibrous tissue forming oil cysts. These lesions are typically surrounded by lipid-laden macrophages, multinucleated giant cells, and histiocytes, reflecting an ongoing reparative response [41].

With disease progression, fibrosis becomes more prominent and may be associated with hemosiderin deposition around areas of necrotic fat and cellular debris. Over time, inflammatory components are replaced by dense fibrotic tissue, resulting in scar formation. Calcifications often develop as a late manifestation of tissue injury and repair following radiotherapy and surgery [42].

### 6.1. Fat Necrosis and Calcifications

Fat necrosis is a benign, non-suppurative inflammatory process of adipose tissue that frequently develops after breast surgery and radiotherapy. Although relatively common in clinical practice, it may represent a diagnostic challenge because of its wide spectrum of imaging appearances, which may range from clearly benign findings to features that closely mimic malignancy [41]. This variability largely reflects the relative proportions of liquefied fat, fibrosis, hemorrhage, and calcifications within the lesion, as well as the temporal evolution of the reparative process [42].

**Figure 4:**
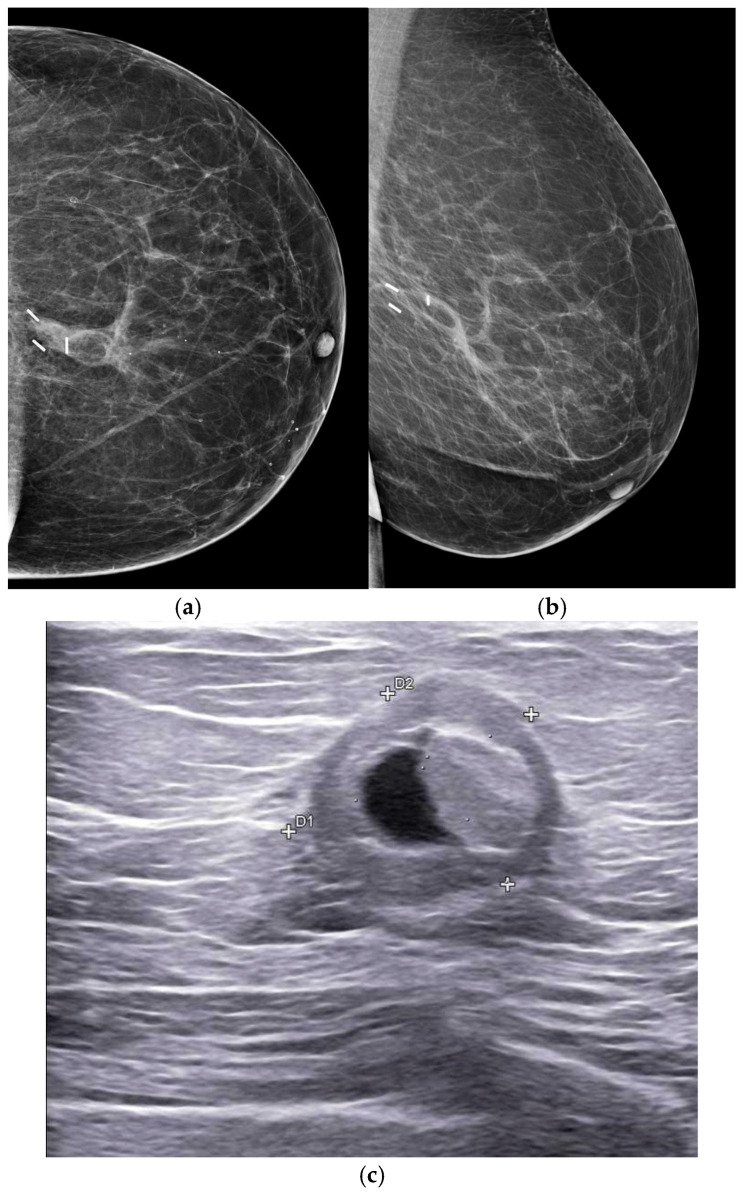
A 58-year-old woman undergoing follow-up at the completion of treatment, one year after surgery and radiotherapy. Features consistent with fat necrosis are present, characterized mammographically by a radiolucent area with thin, peripheral dystrophic calcifications (**a**,**b**). Fat necrosis typically results from adipose tissue injury and evolves through inflammatory and fibrotic stages. On ultrasound, the finding corresponds to a well-defined liponecrotic cyst containing internal echoes or mixed echogenic material, reflecting liquefaction of necrotic fat (**c**).

On mammography, fat necrosis may demonstrate a variety of appearances depending on the degree of fibrosis and the stage of lesion evolution. The most characteristic presentation is that of a radiolucent oil cyst, which represents liquefied fat surrounded by a thin fibrous capsule. Over time, peripheral calcifications may develop along the cyst wall, gradually forming the typical curvilinear or “eggshell” pattern (Figure 4a,b). In other cases, particularly when the fibrotic reaction is more pronounced, fat necrosis may appear as a focal asymmetry, an irregular mass, or an area of architectural distortion. These appearances may occasionally raise suspicion for malignancy, especially when associated with irregular calcifications or increasing density on follow-up examinations.

Ultrasound findings are often variable and may include hyperechoic areas within the subcutaneous tissue, complex cystic lesions with internal echoes, or hypoechoic masses with posterior acoustic shadowing. Hyperechoic lesions are generally considered benign and frequently correspond to fat necrosis (Figure 4c); however, the sonographic appearance may vary considerably depending on the stage of the lesion. Features such as indistinct margins, angular contours, or internal vascularity should be interpreted cautiously and may warrant further investigation or image-guided biopsy when clinical or imaging findings are inconclusive [42,43].

MRI also demonstrates a wide spectrum of appearances. The most typical finding is a lesion with signal intensity similar to fat on both T1- and T2-weighted sequences, often showing signal suppression on fat-saturated images. Additional features may include fat–fluid levels, peripheral rim enhancement, or associated dystrophic calcifications. In many cases, correlation with clinical history, prior imaging, and lesion stability over time allows confident diagnosis. However, when enhancement patterns appear irregular or progressive, further evaluation may be required to exclude tumor recurrence [44,45].

### 6.2. Architectural Distortion

Radiation therapy induces progressive long-term ischemic changes within the breast. Fibroblasts may remain active after treatment completion, leading to continued collagen deposition and ongoing remodeling of breast architecture. This fibrotic process results in persistent volume loss, parenchymal retraction, and stable architectural distortion, most centered on the surgical cavity. Architectural distortion is frequently observed at the lumpectomy site and develops as a consequence of postsurgical scarring and associated fat necrosis.

**Figure 5:**
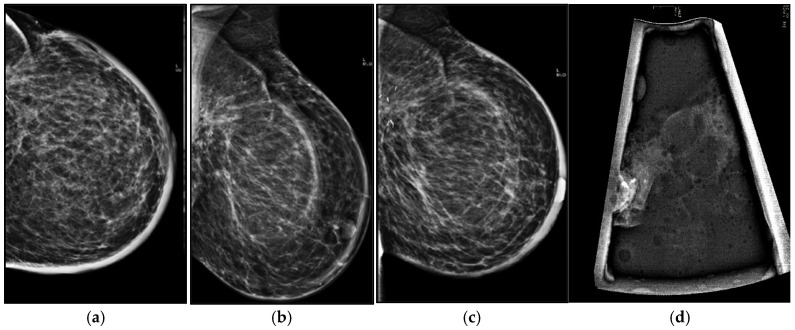
A 59-year-old woman, previously treated with surgery and radiotherapy three years earlier. CC, ML and MLO (**a**–**c**) mammographic views show an extensive area of parenchymal distortion with fibrous retraction of the breast tissue and marked thickening of the skin consistent with post-radiation changes. Within the distorted area, coarse calcific precipitates are visible. Stereotactic vacuum-assisted biopsy was performed, demonstrating calcifications within the sample (**d**) and histological findings of calcific fat necrosis and fibrosis.

On mammography, architectural distortion is typically characterized by central radiolucencies, representing fat entrapped within fibrous scar tissue, associated with thick, curvilinear spiculations. These findings often vary across different projections, reflecting the complex three-dimensional configuration of scar tissue [45]. Although not pathognomonic, such features may aid in distinguishing post-treatment changes from tumor recurrence (Figure 5).

Architectural distortion decreases in prominence over time and then stabilizes on serial examinations. Accordingly, any progressive increase in its extent or conspicuity should raise suspicion for malignancy. Although rare, carcinoma may develop within the surgical scar. In these cases, ultrasound is often of limited value, as it typically demonstrates an irregular hypoechoic mass with posterior acoustic shadowing, an appearance that closely resembles recurrent disease [45].

MRI plays a key role in differentiating postsurgical scar tissue from tumor recurrence. Enhancement within the surgical bed is commonly observed for up to 12 months after treatment completion [46]. Surgical clips and coarse or dystrophic calcifications appear as signal voids. New or increasing enhancement at the postsurgical site beyond 12 months should be considered suspicious and warrants further diagnostic evaluation [46].

## 7. Breast Cancer After Radiotherapy: Recurrence, Secondary Malignancies, and Risk-Adapted Surveillance

Radiotherapy represents a cornerstone in the multidisciplinary management of breast cancer and significantly reduces the risk of ipsilateral breast tumor recurrence, with long-term survival benefits demonstrated in large meta-analyses [5,47]. Although most post-radiotherapy imaging findings reflect benign tissue remodeling, a clinically relevant minority of patients may develop local recurrence or, more rarely, radiation-induced secondary malignancies [48,49]. Accurate differentiation between expected post-treatment changes and true disease progression is therefore essential for optimal patient management.

### 7.1. Local Recurrence

Local recurrence after breast-conserving therapy most commonly arises within or adjacent to the original tumor bed. It results from residual malignant cells that survive surgery and radiotherapy and subsequently regain proliferative potential. In patients treated with breast-conserving surgery and adjuvant radiotherapy, the 10-year cumulative rate of local recurrence is approximately 0.9%, compared with substantially higher rates in patients who do not receive adjuvant irradiation [50]. The risk of recurrence is not uniform and is influenced by several clinicopathological factors, including young age at diagnosis, high tumor grade, lymph node involvement, lymphovascular invasion, close or positive surgical margins, and aggressive molecular subtypes such as triple-negative or HER2-positive disease. Awareness of these risk factors is important when interpreting post-treatment imaging findings and determining the intensity of follow-up. Clinically, recurrence may manifest as focal firmness, new palpable abnormalities, skin retraction, or may remain clinically occult and be detected only on imaging surveillance.

**Figure 6:**
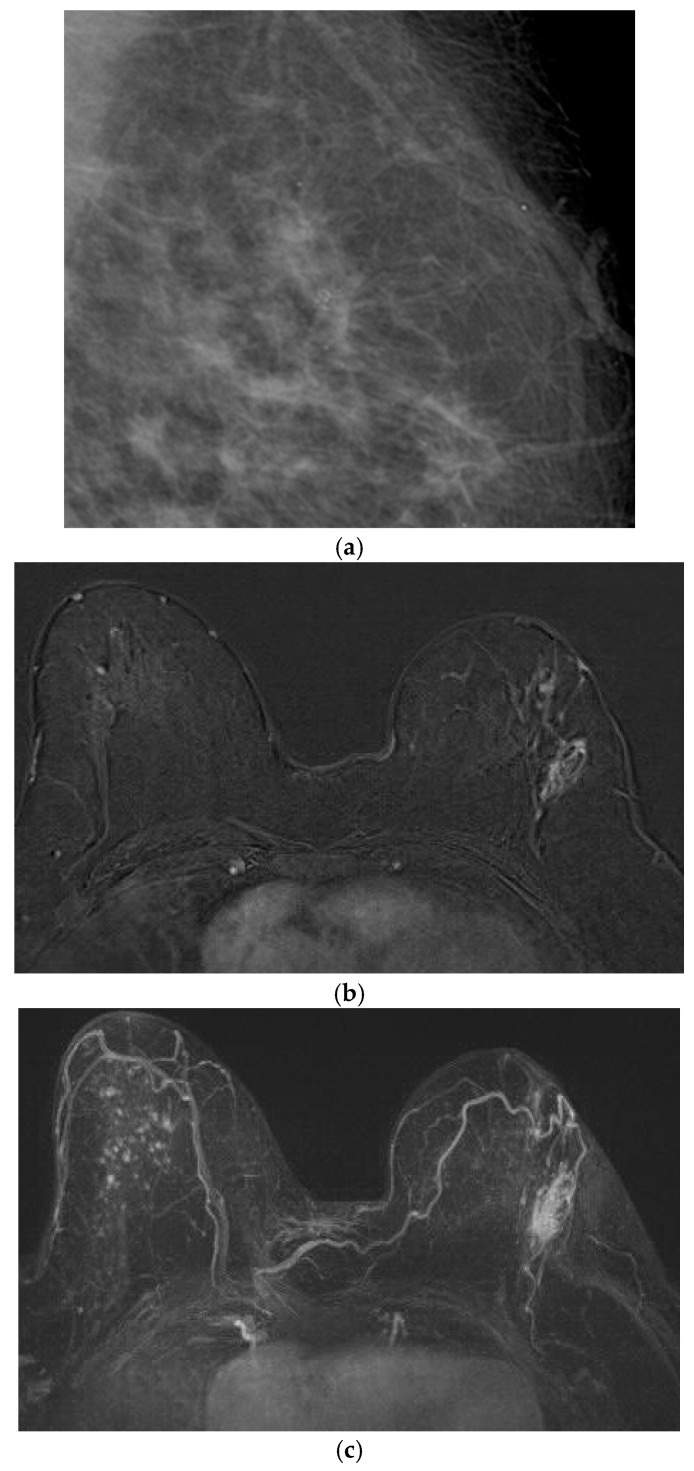
A 64-year-old woman previously treated with breast-conserving surgery and radiotherapy (6 years earlier) shows a new area of parenchymal consolidation with associated pleomorphic calcifications (**a**). Contrast-enhanced T1-weight MRI and MIP reconstructions demonstrate a 2 cm non-mass enhancement located at the site of the surgical scar, findings compatible with disease recurrence (**b**,**c**).

On mammography, recurrent disease typically appears as a new or enlarging spiculated mass, increasing architectural distortion, or suspicious microcalcifications developing within an area of previously stable scar tissue (Figure 6) [51]. Stability over time strongly favors benign post-treatment change, whereas progression after a documented period of stability represents the most important imaging red flag.

Ultrasound usually demonstrates an irregular hypoechoic mass with indistinct or angular margins and internal vascularity. However, significant overlap exists between recurrent tumors and post-radiotherapy fibrosis, particularly within the first year after treatment [51].

Magnetic resonance imaging (MRI) is the most sensitive imaging modality for detecting local recurrence, especially in dense breasts or when conventional imaging findings are equivocal [52]. Recurrent tumors typically demonstrate rapid initial enhancement and washout kinetics. Nevertheless, MRI specificity is highly dependent on timing, as enhancement within the first 6–12 months after radiotherapy may reflect inflammatory or reparative processes rather than viable tumor (Figure 6). Progressive enlargement or increasing enhancement on serial examinations remains the most reliable imaging indicator of recurrence.

When imaging findings are suspicious or demonstrate interval progression, image-guided core biopsy is mandatory for definitive diagnosis.

### 7.2. Radiation-Induced Breast Angiosarcoma (RIBAS)

Radiation-induced breast angiosarcoma (RIBAS) is a rare but aggressive late complication of adjuvant radiotherapy. Its reported incidence is below 0.3%, with most cases occurring after a latency period of 5–10 years following radiation exposure [49].

RIBAS is thought to arise from chronic radiation-induced endothelial damage leading to persistent genomic instability and eventual malignant transformation. Unlike local recurrence, which typically develops within the breast parenchyma near the surgical cavity, RIBAS involves the dermis and subcutaneous tissues within the irradiated field [53]. Clinically, patients often present with subtle bruise-like discoloration, violaceous skin patches, progressive skin thickening, or small nodular dermal lesions. These findings may initially appear benign, contributing to delayed diagnosis. Imaging features are frequently nonspecific. Mammography may show skin thickening or subtle asymmetry without a well-defined mass. Ultrasound may reveal heterogeneous, hypervascular subcutaneous nodules. MRI is generally more informative, demonstrating avidly enhancing dermal or subcutaneous lesions with ill-defined or infiltrative margins [54]. Given the aggressive biological behavior of RIBAS, persistent or progressive dermal abnormalities within a previously irradiated breast—particularly beyond five years after treatment—should prompt early histological confirmation.

### 7.3. Risk Stratification and Tailored Imaging Surveillance

The risk of local recurrence and radiation-induced secondary malignancy is not homogeneous among breast cancer survivors. A risk-adapted surveillance strategy should therefore be considered rather than a uniform follow-up model for all patients. In clinical practice, several patient- and treatment-related factors may influence recurrence risk and the interpretation of post-treatment imaging findings. Tumor biological characteristics, including molecular subtype and tumor grade, patient age at diagnosis, surgical margin status, and lymph node involvement, are well-established determinants of local recurrence risk. In addition, radiotherapy-related parameters such as total dose, delivery of a tumor-bed boost, and irradiated volume may affect both the likelihood of recurrence and the extent of post-treatment imaging changes. These factors should therefore be considered when planning surveillance strategies and when interpreting imaging findings, as patients with higher-risk clinical or pathological features may require closer follow-up and a lower threshold for additional imaging or biopsy compared with low-risk populations.

Patients with high-risk clinicopathological features, including early age at diagnosis, biologically aggressive tumor subtypes, positive lymph nodes, high tumor grade, lymphovascular invasion, or adverse surgical margins—may benefit from closer imaging follow-up and a lower threshold for advanced imaging, particularly MRI, when conventional modalities yield equivocal findings. In selected high-risk patients, some institutions consider alternating annual mammography with MRI during the first 3–5 years after treatment to improve early detection of recurrence [55]. Conversely, in patients with low-risk early-stage disease and stable imaging findings over time, routine annual mammographic surveillance is generally sufficient, provided that no new clinical symptoms emerge. Radiotherapy-related parameters should also be integrated into surveillance planning. Higher total doses, delivery of a tumor-bed boost, larger irradiated volumes, and regional nodal irradiation may influence the extent and persistence of imaging changes, occasionally complicating interpretation [55]. Knowledge of these treatment details improves diagnostic confidence and reduces unnecessary interventions. In long-term survivors, particularly beyond five years after radiotherapy, vigilance for radiation-induced angiosarcoma remains essential. Progressive skin thickening, new dermal nodularity, or increasing vascular subcutaneous lesions should not be attributed solely to chronic radiation effects without appropriate diagnostic work-up. Integration of clinical risk factors, radiotherapy characteristics, and temporal imaging evolution enables a personalized surveillance strategy that balances early detection of clinically significant events with avoidance of unnecessary biopsies and patient anxiety [56].

## 8. Comparative Diagnostic Performance of Imaging Modalities

Beyond the description of individual imaging findings, a comparative perspective on the diagnostic performance of different modalities may further support clinical decision making in the post-radiotherapy setting.

Although multiple imaging modalities contribute to post-radiotherapy surveillance, their diagnostic performance varies depending on clinical context and time from treatment. Mammography remains the cornerstone of routine follow-up because of its reproducibility and high specificity for detecting suspicious calcifications and structural changes. Houssami et al. have reported that mammography maintains high specificity in the surveillance setting but may be limited in patients with complex post-treatment breast anatomy [57]. Ultrasound serves as an important complementary modality, particularly in the evaluation of palpable abnormalities or equivocal mammographic findings although accuracy may be variable because fibrotic scar tissue, fat necrosis, and recurrent tumor can demonstrate overlapping sonographic features. Magnetic resonance imaging demonstrates the highest sensitivity for detecting local recurrence, frequently exceeding 85–95% in selected series showing high negative predictive value and accuracy in differentiating scar tissue from recurrent tumor based on enhancement patterns and temporal evolution [23]. Positron emission tomography/computed tomography is not routinely used for local surveillance, because post-radiotherapy inflammatory uptake may reduce specificity but may play an important role in selected clinical scenarios. PET/CT may be useful when conventional imaging findings are equivocal or when there is suspicion of chest wall recurrence, regional nodal involvement, or distant metastatic disease. In such cases, the whole-body assessment provided by PET/CT may significantly influence clinical management by identifying occult extra-mammary disease or confirming systemic progression [24].

**Figure 7:**
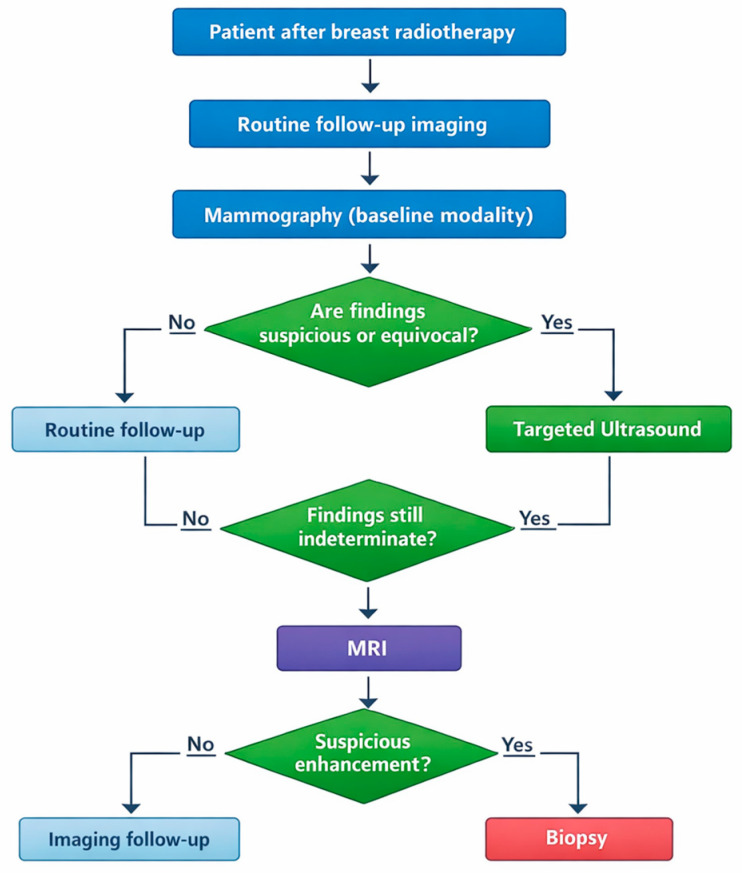
Suggested imaging workflow for post-radiotherapy breast surveillance. Proposed diagnostic algorithm illustrating the sequential use of mammography, targeted ultrasound, and magnetic resonance imaging (MRI) in patients after breast radiotherapy. Mammography represents the baseline modality for routine follow-up, while ultrasound and MRI are used as complementary techniques for the evaluation of suspicious or indeterminate findings. The workflow highlights key decision points that may lead to continued imaging surveillance or image-guided biopsy when malignancy is suspected.

Overall, optimal post-radiotherapy surveillance relies on a multimodal approach that integrates imaging findings, clinical context, radiotherapy parameters, and longitudinal assessment of imaging stability or progression. To facilitate practical application of the imaging strategies discussed in this review, a simplified diagnostic workflow integrating mammography, ultrasound, and MRI in the post-radiotherapy setting is proposed in Figure 7. From a practical perspective, certain imaging findings should prompt a more aggressive diagnostic approach rather than routine follow-up. In general, interval progression after an initial period of stability represents the most important indicator of potential recurrence. New or enlarging masses, increasing architectural distortion, or the development of suspicious calcifications—particularly beyond the first post-treatment year—should prompt further diagnostic evaluation and often image-guided core biopsy.

Conversely, imaging findings that remain stable or gradually regress over time are more likely to represent benign post-treatment changes such as fibrosis, fat necrosis, or postoperative scarring. In cases where imaging findings are indeterminate but lack clearly suspicious features, short-term imaging follow-up may be appropriate.

When discordant findings are observed across different imaging modalities, correlation with clinical history, prior imaging examinations, and knowledge of the surgical and radiotherapy techniques are essential. In selected cases, MRI may provide additional diagnostic information when conventional imaging findings remain equivocal.

## 9. Contemporary Considerations: Systemic Therapy and Reconstruction in the Post-Radiotherapy Setting

Post-radiotherapy imaging interpretation should be considered within the broader context of contemporary multimodal breast cancer management. In recent years, immunotherapy, particularly immune checkpoint inhibitors, has been increasingly incorporated into the treatment of selected breast cancer subtypes, most notably triple-negative disease. Immune-related inflammatory responses may lead to imaging findings such as reactive lymphadenopathy or transient tissue enhancement, which can overlap with post-radiotherapy inflammatory changes and potentially mimic locoregional recurrence. Awareness of systemic treatment history and therapy timing is therefore essential to avoid misinterpretation of immune-related phenomena as disease progression [58].

Breast reconstruction is increasingly performed after mastectomy and may significantly influence the interpretation of post-treatment imaging findings. Both implant-based reconstruction and autologous tissue flaps may alter the normal breast architecture and generate imaging appearances that can occasionally mimic tumor recurrence.

In implant-based reconstruction, the prosthesis typically appears on MRI as a well-defined structure with low signal intensity on T1-weighted sequences and high signal intensity on T2-weighted sequences, surrounded by a fibrous capsule. Following radiotherapy, radiation-induced fibrosis may lead to capsular thickening or capsular contracture, sometimes associated with peri-implant fluid collections or focal periprosthetic soft-tissue thickening. These findings may complicate the differentiation between benign post-treatment changes and recurrent disease [59].

Autologous reconstruction techniques, including transverse rectus abdominis myocutaneous (TRAM) and deep inferior epigastric perforator (DIEP) flaps, introduce adipose and fibrous tissue into the reconstructed breast and are therefore associated with a different spectrum of postsurgical imaging findings including fat necrosis, oil cyst formation, focal fibrosis, and architectural distortion. In some cases, these benign changes may present with irregular margins or enhancement patterns that resemble malignancy [60].

Careful correlation with surgical history, knowledge of the reconstruction technique, and assessment of temporal stability on follow-up examinations are therefore essential to avoid unnecessary biopsies while maintaining sensitivity for the detection of locoregional recurrence.

## 10. Future Perspectives in Post-Radiotherapy Breast Imaging

Recent advances in quantitative imaging and computational analysis are opening new perspectives in the evaluation of post-treatment breast changes. Artificial intelligence (AI)-assisted image analysis is also increasingly being investigated to support radiologists in breast imaging interpretation. In a multi-reader study comparing an AI system with 101 radiologists, Rodriguez-Ruiz et al. reported that the stand-alone AI system achieved cancer detection comparable to that of the average breast radiologist and showed the potential to improve cancer detection rates while maintaining high specificity [61]. These technologies may therefore help identify subtle imaging patterns and reduce interobserver variability in complex post-treatment breast anatomy.

Radiomics approaches allow the extraction of high-dimensional quantitative features from medical images and may provide additional information beyond conventional visual assessment. Lambin and colleagues highlighted the potential of radiomics for developing imaging biomarkers capable of improving tumor characterization and supporting personalized oncologic management [62].

Although these approaches remain largely investigational and are not yet routinely implemented in clinical practice, preliminary evidence suggests that quantitative imaging biomarkers and AI-assisted analysis may contribute in the future to more accurate differentiation between post-radiotherapy fibrosis and tumor recurrence, improving diagnostic objectivity and reproducibility in breast cancer surveillance.

## 11. Conclusions

Radiation therapy induces a broad spectrum of breast tissue changes that evolve over time, ranging from acute inflammatory reactions to chronic fibrotic remodeling. Awareness of the temporal evolution, imaging appearance, and expected progression of these post-treatment changes is essential for accurate interpretation. Although most radiation-related findings are benign and predictable, overlap with the imaging features of local tumor recurrence or, more rarely, radiation-induced malignancies may pose a diagnostic challenge. Comprehensive knowledge of radiation-induced tissue responses, together with careful assessment of atypical or progressive imaging findings, is crucial for early detection of recurrence and for avoiding unnecessary diagnostic or therapeutic interventions, contributing to optimal patient outcomes.

## Figures and Tables

**Table 1:** Common breast radiotherapy schedules: indications, pros/cons, and imaging implications.

Regimen	Typical Prescription	Typical Indications (When Chosen)	Advantages	Limitations/Imaging Implications	Key Evidence/Guideline
Whole-breast irradiation (WBI), conventional	45–50.4 Gy in 25–28 fr (±boost 10–16 Gy)	Less common today; still used when anatomy, prior RT, or institutional practice favors conventional fractionation	Long-standing evidence base; smaller dose per fraction	Longer overall treatment; post-RT edema/skin thickening often prominent early; boost-related focal distortion at lumpectomy bed	ASTRO WBI guideline; EBCTCG meta-analysis [1,2]
WBI, moderate hypofractionation	40–42.5 Gy in 15–16 fr (±boost)	Standard for most patients after BCS, including many node-positive settings	Shorter course; non-inferior local control; comparable or improved cosmesis	Acute changes, similar but compressed timeline; boost increases localized fibrosis/distortion	START A/B; Canadian trial; ASTRO guideline [3,4,5,6]
WBI/chest wall, ultra-hypofractionation (FAST-Forward)	26 Gy in 5 fr over 1 week (selected patients)	Appropriate when meeting FAST-Forward-like criteria; increasingly adopted	Truly short course; non-inferior local control at 5 years	Higher dose per fraction: localized late effects possible; careful correlation with boost use and surgical bed is essential	FAST-Forward trial [7]
Partial-breast irradiation (PBI)	Technique-dependent (e.g., external beam, 38.5 Gy/10 fr BID; brachytherapy; IORT single fraction)	Selected low-risk early-stage disease; typically older age, small tumors, negative margins/nodes	Treats limited volume; reduces exposure to heart/lung; shorter treatment	Imaging changes are more focal and can be striking at cavity; fat necrosis and seroma may be common; requires knowledge of technique	IMPORT LOW; TARGIT-A; ELIOT; guideline criteria [8,9,10]
Regional nodal irradiation (RNI)/post-mastectomy RT	45–50.4 Gy in 25–28 fr or 40–42.4 Gy in 15–16 fr	Node-positive or high-risk features; post-mastectomy chest wall ± nodes; selected neoadjuvant settings	Improves locoregional control in appropriate risk groups	Broader field: skin thickening and edema may extend beyond breast; internal mammary field may affect anterior chest wall; reconstruction complicates MRI interpretation	Randomized/pooled data; contemporary de-escalation trial in ypN0 [11,12,13]

**Table 2:** Time-dependent imaging findings after breast radiotherapy (expected vs. suspicious).

Timeframe	Mammography	Ultrasound	MRI	PET/CT (FDG)	Red Flags Prompting Work-Up
0–6 months	Diffuse skin thickening; increased density; trabecular thickening; seroma/hematoma	Diffuse hypoechoic subcutaneous edema; seroma with variable internal echoes; reactive hyperemia may be present	T2 edema; skin thickening; postoperative seroma; mild, peripheral enhancement can be reactive	Diffuse mild-to-moderate uptake in irradiated tissues	New/enlarging focal mass; rapidly increasing asymmetry; nodular enhancing focus at bed with aggressive kinetics; focal intense FDG uptake not fitting diffuse inflammatory pattern
6–24 months	Evolving fat necrosis/oil cysts; coarse/dystrophic calcifications; stable or decreasing distortion at lumpectomy bed	Hyperechoic areas (fat necrosis); complex cystic lesion; shadowing related to scar/fibrosis	Fat-signal lesions with minimal/no internal enhancement; decreasing enhancement at surgical bed	Variable uptake depending on active inflammation vs. fibrosis	Pleomorphic/linear branching calcifications; enlarging spiculated mass; increasing distortion; new nodular or mass-like enhancement, especially if progressive on serial exams
>24 months	Stable scar and calcifications; overall stabilization of density and skin thickness	Stable shadowing/scar; stable fat necrosis	Minimal stable enhancement; stable distortion; reconstruction-related changes if applicable	Uptake should be low/stable in absence of disease	Any new or progressive finding; new enhancement within/adjacent to bed; new suspicious calcifications; progressive distortion

## Data Availability

No new data were created or analyzed in this study.
